# Dexamethasone versus methylprednisolone for multiple organ dysfunction in COVID-19 critically ill patients: a multicenter propensity score matching study

**DOI:** 10.1186/s12879-024-09056-y

**Published:** 2024-02-13

**Authors:** Ohoud Aljuhani, Ghazwa B. Korayem, Ali F. Altebainawi, Daniah AlMohammady, Amjaad Alfahed, Elaf F. Altebainawi, Mohammed Aldhaeefi, Hisham A. Badreldin, Ramesh Vishwakarma, Faisal E. Almutairi, Abeer A. Alenazi, Thamer Alsulaiman, Rahaf Ali Alqahtani, Fahad Al Dhahri, Namareq Aldardeer, Ahmed O. Alenazi, Shmeylan Al Harbi, Raed Kensara, Mai Alalawi, Khalid Al Sulaiman

**Affiliations:** 1 Department of Pharmacy Practice, Faculty of Pharmacy, King Abdulaziz University, Jeddah, Saudi Arabia; 2 Department of Pharmacy Practice, College of Pharmacy, Princess Nourah bint Abdulrahman University, Riyadh, Saudi Arabia; 3 Pharmaceutical Care Services, King Salman Specialist Hospital, Hail Health Cluster, Ministry of Health, Hail, Saudi Arabia; 4 Department of Medicine, King Khalid Hospital, Hail Health Cluster, Hail, Saudi Arabia; 5 Pharmaceutical Care Department, King Abdulaziz Medical City, Riyadh, Saudi Arabia; 6 College of Pharmacy, King Saud Bin Abdulaziz University for Health Sciences, Riyadh, Saudi Arabia; 7 King Abdullah International Medical Research Center-King Saud Bin Abdulaziz University for Health Sciences, Ministry of National Guard – Health Affairs, Riyadh, Saudi Arabia; 8 Norwich clinical trial unit, Norwich medical school, University of east Anglia, Norwich, UK; 9 Clinical Pharmacy Department, Pharmacy Services Administration, King Fahad Medical City, Riyadh, Saudi Arabia; 10 Pharmaceutical Care Department, Prince Sultan Military Medical City, Riyadh, Saudi Arabia; 11 Family Medicine Department, King Faisal Specialist Hospital & Research Center, Riyadh, Saudi Arabia; 12 Pharmaceutical Care Services, King Faisal Specialist Hospital and Research Center, Jeddah, Saudi Arabia; 13 Pharmaceutical Care Department, King Abdulaziz Medical City, Dammam, Saudi Arabia; 14 Pharmaceutical Care Department, King Abdulaziz Medical City, Jeddah, Saudi Arabia; 15 Pharmaceutical Care Services, King Abdulaziz Medical City, Jeddah, Saudi Arabia; 16 Clinical and Administrative Pharmacy Sciences, College of Pharmacy, Howard University, Washington, DC, 20059 USA; 17 Department of Clinical Pharmacy, College of Pharmacy, University of Hail, Hail, Saudi Arabia; 18 King Abdulaziz Medical City (KAMC) - Ministry of National Guard Health Affairs (MNGHA), King Abdullah International Medical Research Center/King Saud bin Abdulaziz University for Health Sciences, PO Box 22490, Riyadh, 11426 Saudi Arabia

**Keywords:** COVID-19, SARS-Cov-2, Dexamethasone, Methylprednisolone, Critically ill, ICUs, 30-day mortality, In-hospital mortality, Ventilation free days (VFDs)

## Abstract

**Background:**

Dexamethasone usually recommended for patients with severe coronavirus disease 2019 (COVID-19) to reduce short-term mortality. However, it is uncertain if another corticosteroid, such as methylprednisolone, may be utilized to obtain better clinical outcome. This study assessed dexamethasone’s clinical and safety outcomes compared to methylprednisolone.

**Methods:**

A multicenter, retrospective cohort study was conducted between March 01, 2020, and July 31, 2021. It included adult COVID-19 patients who were initiated on either dexamethasone or methylprednisolone therapy within 24 h of intensive care unit (ICU) admission. The primary outcome was the progression of multiple organ dysfunction score (MODS) on day three of ICU admission. Propensity score (PS) matching was used (1:3 ratio) based on the patient’s age and MODS within 24 h of ICU admission.

**Results:**

After Propensity Score (PS) matching, 264 patients were included; 198 received dexamethasone, while 66 patients received methylprednisolone within 24 h of ICU admission. In regression analysis, patients who received methylprednisolone had a higher MODS on day three of ICU admission than those who received dexamethasone (beta coefficient: 0.17 (95% CI 0.02, 0.32), *P* = 0.03). Moreover, hospital-acquired infection was higher in the methylprednisolone group (OR 2.17, 95% CI 1.01, 4.66; *p* = 0.04). On the other hand, the 30-day and the in-hospital mortality were not statistically significant different between the two groups.

**Conclusion:**

Dexamethasone showed a lower MODS on day three of ICU admission compared to methylprednisolone, with no statistically significant difference in mortality.

**Supplementary Information:**

The online version contains supplementary material available at 10.1186/s12879-024-09056-y.

## Introduction

Severe acute respiratory syndrome coronavirus 2 (SARS-CoV-2) is the leading cause of coronavirus disease 2019 (COVID-19) [1]. Around 26–32% of patients with COVID-19 may require intensive care unit (ICU) admission [2, 3]. The mortality rate of critical COVID-19 cases ranges from 26%-48% [4–6]. Patients infected with SARS-CoV-2 experience a systemic hyperinflammatory response [7, 8]. This reaction is driven by the high cytokine levels, causing several life-threatening complications such as acute respiratory distress syndrome and multisystem organ failure [7, 8]. The immunomodulatory agents targeting cytokine release and hyperinflammatory response have been a potential treatment option for critically ill patients with COVID-19 [9, 10]. Several immunomodulators agents, such as corticosteroids, interleukin-6 inhibitors (IL-6), and Janus kinase (JAK) inhibitors, have been investigated to treat hospitalized patients with COVID-19 [11–14].

Corticosteroids have anti-inflammatory and immunosuppressive actions that reduce the production of pro-inflammatory cytokines such as IL-6 [15]. Previous studies have shown improved prognosis and survival rate in patients with moderate to severe COVID-19 using corticosteroids therapy, including dexamethasone, methylprednisolone, and hydrocortisone [10, 14, 16–18]. However, the use of hydrocortisone or methylprednisolone in this patients population was not supported by solid evidence [19]. The RECOVERY trial is a randomized control study that included 6425 hospitalized patients with COVID-19. The study found that patients using dexamethasone along with standard therapy had a lower mortality rate at 22.9% compared to patients who received standard treatment alone at 25.7% (rate ratio, 0.83; 95% confidence interval (CI), 0.75 to 0.93; *P* < 0.001) [19].

Methylprednisolone had greater lung tissue-to plasma penetration in rats when compared to dexamethasone. This effect of methylprednisolone might be beneficial to reduce lung damage and prevent respiratory complications [20]. Studies investigating methylprednisolone in patients with severe COVID-19 showed improved survival [10]. Another randomized controlled trial (RCT), including 68 hospitalized patients admitted to the ICU with COVID-19, compared pulse methylprednisolone to the standard of care. This study found the administration of pulse methylprednisolone at the early phase of illness significantly increased survival compared to standard of care (HR 0.293; 95% CI 0.154–0.556; *p* < 0.001). The use of methylprednisolone helped improve pulmonary symptoms and inflammatory markers [16].

Both intravenous methylprednisolone and dexamethasone, administered in a 2 mg/kg/day and 6 mg/day dose, respectively, demonstrated similar efficacy in reducing hospital length of stay and oxygen therapy needs in patients with severe viral infection manifestation [21]. In contrast, a small RCT that enrolled 86 critically ill ventilated patients with COVID-19; found that patients receiving methylprednisolone have shorter hospitalization and less dependency on mechanical ventilation (MV) compared to dexamethasone [22]. In non-critically ill COVID-19 patients, pneumonia treated with high-dose methylprednisolone showed lower C-reactive protein (CRP), and D-dimer compared to high-dose dexamethasone [1].

Multiple organ dysfunction score (MODS) is a validated tool that measures the severity of MOD in the intensive care unit. MODS consisted of 24 scores that could be used as a prognostic indicator if calculated at ICU admission or as an outcome once calculated during ICU stay. Higher MODS are indicative of a higher probability of ICU mortality [23]. During the pandemic, severe COVID-19 infection was associated with MOD involving cardiovascular, renal, and pulmonary systems [24]. The involvement of many vital organs in patients with coronavirus infection made the MODS as the most relevant indicator for a patient’s complex condition. National Institutes of Health Guidelines recommended using systemic corticosteroids, including dexamethasone and methylprednisolone for hospitalized patients with COVID-19 on supplementary oxygen [19]. Most previous studies investigated the survival benefits and the need for MV. Corticosteroids reduced the risk of endotracheal intubation as indicative of improved lung function [25]. However, the corticosteroid of choice for better clinical outcomes in critically ill patients with COVID-19 remains undetermined. Thus, we conducted this study to assess the short termclinical and safety outcomes of dexamethasone compared to methylprednisolone in critically ill patients with COVID-19.

## Methods

### Study design

This research is a component of the Saudi Critical Care Pharmacy Research (SCAPE) platform, which has conducted numerous investigations into the safety and efficacy of diverse treatments and therapies for critically ill patients (Saudi Critical Care Pharmacy Research (SCAPE), 2023). The present study’s design and methodologies draw upon those employed in our previously published research [6]. This is a multicenter retrospective cohort study. It included adult critically ill patients with confirmed COVID-19 admitted to the ICUs between March 01, 2020, and July 31, 2021. COVID-19 was diagnosed using Reverse Transcriptase-Polymerase Chain Reaction (RT-PCR) nasopharyngeal or throat swabs. We categorized the included patients into two groups depending on the type of corticosteroids used within 24 h of ICU admission (Dexamethasone or Methylprednisolone). We followed eligible patients until hospital discharge or death. The King Abdullah International Medical Research Center (KAIMRC) authorized the study in February 2022 (Ref.# NRC22R-074–02). Due to the study’s retrospective observational nature, informed consent from study participants was waived. All methods were performed following relevant guidelines and regulations.

### Study participants

**Fig. 1:**
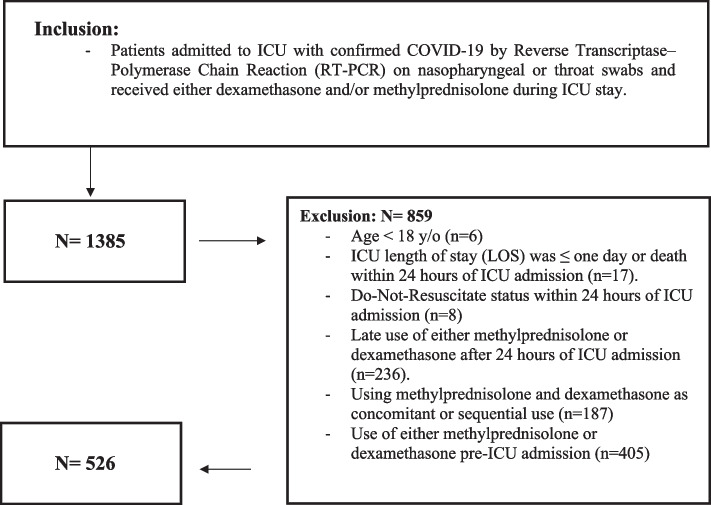
Eligibility criteria flowchart

All adult patients (age ≥ 18 years) with confirmed COVID-19 admitted to the ICUs during the study period who received either dexamethasone or methylprednisolone were assessed for eligibility. Patients were excluded if they received dexamethasone or methylprednisolone before ICU admission or those who were initiated on corticosteroids more than 24 h after ICU admission. Other exclusion criteria include patients who used corticosteroids as concomitant or sequential therapy, ICU discharge, or death within the first 24 h of ICU admission, and those labeled as “Do-Not-Resuscitate” (Fig. 1).

### Study setting

This study was conducted at five hospitals in Saudi Arabia: King Abdulaziz Medical City (Riyadh and Jeddah), King Abdulaziz University Hospital (Jeddah), King Abdullah bin Abdulaziz University Hospital (Riyadh), and King Salman Specialist Hospital (Hail). The selection of these centers was based on the geographic distribution, availability of electronic records, and the center’s willingness to participate in the national project. The primary site for this multicenter study was King Abdulaziz Medical City (KAMC- Riyadh), a tertiary care center.

### Data collection

Each patients’ data were collected and managed using Research Electronic Data Capture (REDCap®) software hosted by KAIMRC. We gathered patients’ demographic data, comorbidities, vital signs, laboratory tests, baseline severity scores (i.e., MODS, Acute Physiology and Chronic Health Evaluation II (APACHE II), and Sequential Organ Failure Assessment (SOFA)), renal profile, acute kidney injury, use of prone positioning, MV use and parameters (e.g., PaO2/FiO2 ratio, FiO2 requirement) within 24 h of ICU admission. The Glasgow Coma Score (GCS) was collected in the study, with sedation assessment using the Richmond Agitation-Sedation Scale (RASS) for mechanically ventilated (MV) patients. Conscious level evaluation for non-MV patients or those on non-invasive MV utilized the GCS score. Verbal scores were excluded in invasively intubated and sedated patients due to their inability to vocalize, and assessment focused on eye opening and motor scores, allowing for the computation of the GCS sum score in intubated patients. For noninvasive MV patients, the best GCS was used for conscious level assessment, and for invasive MV patients, GCS scores were measured during sedation vacations, offering a comprehensive understanding of their neurological status. We gathered information on liver function tests (LFTs), coagulation profile (i.e., INR, aPTT, fibrinogen, D-dimer), and inflammatory markers (Ferritin, procalcitonin, and creatine phosphokinase (CPK)) within 24 h of ICU admission. Data on the use of corticosteroids therapy (type, dose, and duration), the timing of corticosteroids initiation, and tocilizumab use were collected for the eligible patients.

### Outcomes

The primary outcome was the multiple organ dysfunction on day three of ICU admission. The secondary outcomes were mortality, hospital Length of Stay, ICU Length of Stay, respiratory failure requiring MV, ventilator-free days (VFDs) at 30 days, and ICU-acquired complication**s** (new-onset atrial fibrillation, AKI, liver injury, hospital-acquired infection, pneumonia, and secondary fungal infection). Outcomes definitions are shown in the Additional file 1.

### Statistical analysis

We presented continuous vaiables as mean and standard deviation (SD), or median and lower quartile (Q1) and upper quartile (Q3), as appropriate and categorical variables as number (percentage). The normality assumptions were assessed for all numerical variables using a statistical test (i.e., Shapiro–Wilk test) and graphical representation (i.e., histograms and Q-Q plots). Model fit was assessed using the Hosmer–Lemeshow goodness-of-fit test.

Baseline characteristics and outcome variables were compared between the two study groups. We used the Chi-square or Fisher’s exact test for categorical variables. We compared the normally distributed continuous variables using the student t-test and other non-normally distributed continuous variables with the Mann–Whitney U test. Multivariable Cox proportional hazards regression analyses were performed for the 30-day and in-hospital mortality. The proportionality assumption was assessed before fitting the cox model. Visual assessment was performed to evaluate the hypothesis by plotting a log(-log) plot and testing the correlation of scaled Schoenfeld residuals with rank-ordered time.

Multivariable logistic regression analysis and negative binomial regression were used for the other outcomes considered in this study. Regression analysis was done for the study outcomes after considering propensity scores (PS) as covariates in the model. These scores are generated through PS analysis after considering all relevant covariates, which includes patient’s age and MODS within 24 h of ICU admission. The odds ratios (OR), hazard ratio (HR), or estimates with the 95% CI were reported as appropriate. No imputation was made for missing data as the cohort of patients in our study was not derived from random selection.

The Proc PS match procedure (SAS Cary, NC) was used to match patients who received methylprednisolone therapy (active group) to patients who received dexamethasone therapy (control group) (1:3 ratio) based on patient’s age and MODS within 24 h of ICU admission. A greedy nearest neighbor matching method was utilized; one patient who received methylprednisolone therapy (active) paired with three patients who received dexamethasone (control group), which eventually produced the smallest within-pair difference among all available pairs with treated patients. The difference in the logits of the PS for pairs of patients from the two groups was matched if it was less than or equal to 0.5 times the pooled estimate of the standard deviation. We considered a *P* value of < 0.05 statistically significant and used SAS version 9.4 for all statistical analyses.

## Results

Among 1385 patients who were screened during the study period, 526 patients enrolled after applying the study exclusion criteria. Of these patients, 455 were given dexamethasone, while 71 were given methylprednisolone therapy 24 h after ICU admission. After PS matching (1:3), 264 patients were included (66 patients received methylprednisolone, while 198 patients received dexamethasone) according to the selected criteria. The median dose of methylprednisolone and dexamethasone per day was 80 mg (80, 120) and 6 mg (6, 6), respectively. Methylprednisolone was administered for a median duration of six days (4.7, 10) compared with ten days (9, 11) for the dexamethasone group.

### Demographic and clinical characteristics

Most of the patients were male (65.8%) in the whole cohort, and the average age was 62.0 ± 15.25 years. Diabetes mellitus (60.0%) was the most common comorbidity, followed by hypertension (56.3%), dyslipidemia (21.9%), and chronic kidney disease (11.2%). There was a statistically significant difference in the baseline characteristics between the two groups before PS matching.

Patients who received dexamethasone have a lower PaO2/FiO2 ratio, higher use of inotropes/vasopressors, and GCS at admission than methylprednisolone. After PS matching based on the patient’s age and MODS within 24 h of ICU admission, most of the baseline demographics and clinical characteristics were well balanced between the two groups, except for the higher total bilirubin, INR, and P/F ratio in the methylprednisolone group. There were no significant differences in severity scores within 24 h of admission (APACHE II, SOFA scores, and MODS) between the two groups, regardless of PS matching. In addition, the proportion of tocilizumab use within 24 h of ICU admission and the concomitant use of nephrotoxic medications during ICU stay were similar between the two groups (Table 1).

**Table 1:** Summary of demography and baseline characteristics

	**Before propensity score (PS)**				**After propensity score (PS)**			
	**Overall (*N* = 526)**	**Dexamethasone (*N* = 455)**	**Methylprednisolone (*N* = 71)**	***P*-value**	**Overall (*N* = 264)**	**Dexamethasone (*N* = 198)**	**Methylprednisolone (*N* = 66)**	***P*-value**
**Age (Years), Mean (SD)**	62.0 (15.25)	62.3 (14.92)	60.2 (17.14)	0.3336^^^	59.7 (15.77)	59.6 (15.52)	60.1 (16.60)	0.9088^^^
**Gender – Male, n (%)**	336 (65.8)	298 (67.6)	38 ( 54.3)	0.0295^^^^	168 (64.6)	131 (67.5)	37 (56.1)	0.0924^^^^
**Weight (kg), Mean (SD)**	81.2 (18.82)	80.8 (18.70)	83.2 (19.51)	0.2456^^^	82.6 (21.03)	82.4 (21.58)	83.4 (19.48)	0.5130^^^
**APACHE II score, Median (Q1,Q3)**	14.0 (9.00, 22.00)	14.0 (9.00, 22.00)	15.0 (9.00, 22.00)	0.6307^^^	15.0 (10.00, 22.00)	15.0 (10.00, 24.00)	15.0 (9.00, 21.00)	0.7147^^^
**SOFA score, Median (Q1,Q3)**	4.0 (2.00, 6.00)	4.0 (2.00, 6.00)	5.0 (2.00, 9.00)	0.0808^^^	5.0 (3.00, 8.00)	5.0 (3.00, 8.00)	5.0 (2.00, 7.00)	0.3863^^^
**Multiple Organ Dysfunction Score at admission, Median (Q1,Q3)**	5.0 (4.00, 7.00)	5.0 (4.00, 7.00)	6.0 (4.00, 9.00)	0.0821^^^	6.0 (4.00, 8.00)	5.0 (4.00, 8.00)	6.0 (4.00, 9.00)	0.6028^^^
**Early use of Tocilizumab within 24 h, n (%)**	104 (20.1)	92 (20.6)	12 (16.9)	0.4669^^^^	53 (20.2)	41 (20.9)	12 (18.2)	0.6322^^^^
**Proning at admission, n (%)**	129 (25.5)	115 (26.4)	14 (20.0)	0.2559^^^^	57 (22.4)	45 (23.7)	12 (18.5)	0.3830^^^^
**Serum creatinine (mmol/L) at admission, Median (Q1,Q3)**	89.4 (69.00, 135.00)	88.0 (69.00, 130.40)	98.5 (66.00, 148.00)	0.5853^^^	90.0 (68.00, 138.00)	88.0 (68.00, 138.00)	96.3 (69.00, 141.00)	0.6705^^^
**Blood Urea nitrogen (BUN) at admission, Median (Q1,Q3)**	7.1 (4.80, 12.00)	7.1 (4.80, 11.60)	6.9 (4.70, 12.30)	0.9834^^^	7.2 (4.80, 12.80)	7.2 (4.80, 12.80)	6.9 (4.83, 13.30)	0.9097^^^
**Acute Kidney Injury (AKI) within 24 h of ICU admission, n (%)**	157 (30.5)	132 (29.7)	25 (36.2)	0.2703^^^^	84 (32.3)	61 (31.1)	23 (35.9)	0.4745^^^^
**Mechanical Ventilation within 24 h of ICU admission, n (%)**	378 (73.4)	329 (74.1)	49 (69.0)	0.3679^^^^	196 (75.4)	151 (77.8)	45 (68.2)	0.1158^^^^
**Oxygenation Index (OI), Median (Q1,Q3)**	16.3 (8.40, 28.19)	17.7 (8.12, 28.50)	13.8 (9.10, 24.28)	0.5501^^^	15.0 (7.89, 27.73)	19.6 (7.09, 28.50)	13.2 (9.10, 23.78)	0.5762^^^
**Inotropes/vasopressors use within 24 h of admission), n(%)**	129 (25.1)	102 (23.1)	27 (38.0)	0.0070^^^^	84 (32.3)	59 (30.4)	25 (37.9)	0.2625^^^^
**Vasoactive Inotropic Score, Mean (SD)**	9.1 (48.92)	7.8 (42.12)	18.3 (81.79)	0.1088^^^	12.8 (59.78)	11.1 (50.05)	18.2 (84.20)	0.7957^^^
**Lactic acid Baseline, Median (Q1,Q3)**	1.7 (1.29, 2.40)	1.7 (1.29, 2.37)	1.8 (1.26, 2.43)	0.8597^^^	1.6 (1.21, 2.37)	1.6 (1.20, 2.26)	1.8 (1.26, 2.47)	0.6409^^^
**Platelets count Baseline, Median (Q1,Q3)**	244.0 (190.00, 319.00)	244.0 (189.00, 319.00)	243.0 (196.00, 319.00)	0.7916^^^	241.0 (189.00, 314.00)	241.0 (178.00, 318.00)	239.0 (199.00, 314.00)	0.5410^^^
**Total WBC Baseline, Median (Q1,Q3)**	9.7 (6.64, 12.70)	9.6 (6.64, 12.71)	9.8 (6.35, 12.40)	0.8832^^^	9.8 (6.81, 13.35)	9.4 (6.81, 13.90)	10.0 (6.81, 12.70)	0.9539^^^
**International normalized ratio (PT-INR), Median (Q1,Q3)**	1.1 (1.01, 1.20)	1.1 (1.01, 1.19)	1.1 (1.02, 1.23)	0.2059^^^	1.1 (1.01, 1.17)	1.1 (1.01, 1.14)	1.1 (1.03, 1.23)	0.0362^^^
**activated partial thromboplastin time (aPTT) Baseline, Median (Q1,Q3)**	30.5 (27.10, 34.00)	30.6 (27.50, 34.00)	29.2 (25.80, 34.00)	0.0797^^^	30.5 (26.90, 34.00)	30.7 (27.50, 34.00)	29.0 (25.60, 34.00)	0.0910^^^
**Alanine transaminase (ALT) at admission, Median (Q1,Q3)**	38.0 (24.00, 59.50)	39.0 (24.00, 58.00)	35.0 (25.00, 64.00)	0.9669^^^	36.5 (23.50, 60.00)	37.0 (23.00, 58.00)	35.5 (26.00, 64.00)	0.7618^^^
**Aspartate transaminase (AST) at admission, Median (Q1,Q3)**	49.0 (34.00, 77.00)	50.0 (34.00, 78.00)	43.0 (32.00, 74.00)	0.4236^^^	48.0 (33.00, 76.00)	49.0 (35.00, 77.00)	43.5 (32.00, 74.00)	0.4346^^^
**Albumin Baseline, Median (Q1,Q3)**	33.0 (29.00, 36.00)	33.0 (29.00, 36.00)	31.0 (28.00, 35.00)	0.1400^^^	33.0 (29.00, 36.00)	33.0 (29.00, 36.00)	31.0 (28.00, 35.00)	0.0754^*^
**Hematocrit at admission, Median (Q1,Q3)**	0.4 (0.34, 0.43)	0.4 (0.34, 0.43)	0.4 (0.33, 0.44)	0.4434^^^	0.4 (0.33, 0.44)	0.4 (0.34, 0.44)	0.4 (0.32, 0.44)	0.2633^^^
**Creatine phosphokinase (CPK) baseline (U/l), Median (Q1,Q3)**	169.5 (74.00, 428.00)	164.0 (74.00, 403.00)	272.0 (88.00, 598.00)	0.0729^^^	183.0 (83.00, 528.00)	180.5 (82.00, 472.00)	282.0 (84.00, 638.00)	0.2966^^^
**C-reactive protein (CRP) baseline (mg/l), Median (Q1,Q3)**	138.0 (81.30, 204.12)	137.0 (81.00, 200.00)	163.5 (82.50, 246.00)	0.2152^^^	138.0 (83.00, 209.00)	135.0 (84.00, 201.50)	168.0 (82.50, 248.00)	0.1595^^^
**Fibrinogen Level baseline (gm/l), Median (Q1,Q3)**	5.5 (3.97, 7.25)	5.5 (3.96, 7.27)	5.7 (4.03, 7.22)	0.6693^^^	6.1 (4.06, 7.56)	6.1 (4.05, 7.68)	6.0 (4.64, 7.22)	0.8279^^^
**D-dimer Level baseline, Median (Q1,Q3)**	1.5 (0.77, 3.70)	1.5 (0.76, 3.38)	2.0 (0.95, 4.31)	0.0705^^^	1.6 (0.77, 4.06)	1.5 (0.71, 3.43)	2.4 (0.95, 4.31)	0.0581^^^
**Ferritin Level baseline, Median (Q1,Q3)**	699.0 (365.00, 1509.44)	710.7 (364.08, 1509.44)	672.5 (387.00, 1741.10)	0.8613^^^	824.2 (415.70, 2026.40)	877.1 (427.60, 2115.50)	709.1 (389.20, 1741.10)	0.4454^^^
**Blood glucose level Baseline Within 24 h of ICU admission, Median (Q1,Q3)**	11.3 (8.00, 16.00)	11.2 (8.30, 16.10)	12.0 (7.30, 16.00)	0.7540^^^	11.2 (7.90, 16.15)	11.0 (8.00, 16.30)	12.5 (7.40, 16.00)	0.8602^^^
**Best GCS at admission, Median (Q1,Q3)**	15.0 (13.00, 15.00)	15.0 (14.00, 15.00)	15.0 (11.00, 15.00)	0.0020^^^	15.0 (11.00, 15.00)	15.0 (10.00, 15.00)	15.0 (11.00, 15.00)	0.1759^^^
**PaO2/FiO2 ratio within 24 h of admission, Median (Q1,Q3)**	81.5 (59.70, 132.83)	79.4 (58.33, 124.67)	108.6 (66.88, 183.50)	0.0035^^^	86.9 (61.68, 148.00)	80.6 (60.12, 132.83)	113.1 (67.04, 183.97)	0.0119^^^
**FIO2 requirement (%) at admission, Median (Q1,Q3)**	75.0 (50.00, 100.00)	75.0 (50.00, 100.00)	65.0 (45.00, 100.00)	0.1286^^^	75.0 (50.00, 100.00)	80.0 (50.00, 100.00)	65.0 (42.50, 97.50)	0.1175^^^
**Respiratory rate (Breath Per Minute) at admission, Median (Q1,Q3)**	28.0 (23.00, 33.00)	28.0 (23.00, 33.00)	27.5 (23.00, 32.00)	0.9705^^^	28.0 (23.00, 32.50)	28.0 (23.00, 33.00)	27.5 (23.00, 32.00)	0.9992^^^
**Highest heart rate (HR) at admission, Median (Q1,Q3)**	102.0 (90.00, 114.00)	101.0 (90.00, 114.00)	104.0 (94.00, 115.00)	0.1478^^^	103.0 (89.00, 114.00)	101.0 (86.50, 114.00)	104.0 (94.00, 114.00)	0.2449^^^
**Lowest MAP at admission, Median (Q1,Q3)**	73.8 (63.00, 84.00)	73.7 (64.00, 84.00)	74.0 (63.00, 83.00)	0.5126^^^	71.0 (61.00, 80.00)	70.0 (60.50, 79.50)	73.0 (63.00, 83.00)	0.3186^*^
**Maximum body temperature Baseline, Median (Q1,Q3)**	37.2 (37.00, 38.00)	37.2 (37.00, 38.00)	37.3 (37.00, 38.10)	0.4238^^^	37.2 (37.00, 38.00)	37.2 (37.00, 38.00)	37.2 (37.00, 38.10)	0.6709^^^
**Pharmacological DVT prophylaxis use during ICU stay,n (%)**	468 (90.7)	404 (90.6)	64 (91.4)	0.8209^^^^	242 (92.7)	182 (92.9)	60 (92.3)	0.8825^**^
High dose of Pharmacological DVT prophylaxis, n(%)^b^	195 (41.8)	172 (42.6)	23 (36.5)	0.5082^^^^	97 (40.2)	75 (41.2)	22 (37.3)	0.7488^^^^
Standard dose of Pharmacological DVT prophylaxis, n(%)^b^	243 (52.0)	206 (51.0)	37 (58.7)	0.5082^^^^	129 (53.5)	95 (52.2)	34 (57.6)	0.7488^^^^
Low dose of Pharmacological DVT prophylaxis, n(%)^b^	29 (6.2)	26 (6.4)	3 (4.8)	0.5082^^^^	15 (6.2)	12 (6.6)	3 (5.1)	0.7488^^^^
**Patient received nephrotoxic drugs/material during ICU stay, n (%)**^a^	432 (84.0)	374 (84.4)	58 (81.7)	0.5591^^^^	223 (85.4)	169 (86.7)	54 (81.8)	0.3344^^^^
**Comorbidity, n (%)**								
Atrial fibrillation (A Fib)	16 (3.1)	13 (2.9)	3 (4.2)	0.5537^**^	6 (2.3)	3 (1.5)	3 (4.5)	0.1567^**^
Heart Failure	38 (7.4)	33 (7.4)	5 (7.0)	0.9148^^^^	22 (8.4)	17 (8.7)	5 (7.6)	0.7809^^^^
Hypertension	291 (56.3)	255 (57.2)	36 (50.7)	0.3073^^^^	142 (54.2)	108 (55.1)	34 (51.5)	0.6130^^^^
Diabetes Mellitus	310 (60.0)	276 (61.9)	34 (47.9)	0.0254^^^^	150 (57.3)	117 (59.7)	33 (50.0)	0.1686^^^^
Dyslipidemia	113 (21.9)	97 (21.7)	16 (22.5)	0.8816^^^^	60 (22.9)	44 (22.4)	16 (24.2)	0.7642^^^^
Ischemic heart disease (IHD)	35 (6.8)	33 (7.4)	2 (2.8)	0.1535^**^	8 (3.1)	6 (3.1)	2 (3.0)	0.9899^**^
Chronic kidney disease (CKD)	58 (11.2)	49 (11.0)	9 (12.7)	0.6752^^^^	32 (12.2)	23 (11.7)	9 (13.6)	0.6832^^^^
Deep Vein Thrombosis (DVT)	2 (0.4)	2 (0.4)	0 (0.0)	0.5718^**^	1 (0.4)	1 (0.5)	0 (0.0)	0.5610^**^
Pulmonary Embolism (PE)	4 (0.8)	3 (0.7)	1 (1.4)	0.5110^**^	2 (0.8)	1 (0.5)	1 (1.5)	0.4172^**^
Liver disease (any type)	8 (1.5)	8 (1.8)	0 (0.0)	0.2554^**^	4 (1.5)	4 (2.0)	0 (0.0)	0.2422^**^
Stroke	22 (4.3)	19 (4.3)	3 (4.2)	0.9893^**^	13 (5.0)	10 (5.1)	3 (4.5)	0.8571^**^

^*^T Test

^^^Wilcoxon rank sum test is used to calculate the *P*-value

^^^^Chi square

^**^Fisher’s exact teat is used to calculate *P*-value

^a^Nephrotoxic medications/ material included IV Vancomycin, Gentamicin, Amikacin, Contrast, Colistin, Furosemide, and/or Sulfamethoxazole/trimethoprim

^b^Patients who received either Enoxaparin 40 mg daily or UFH 5000 Unit three times daily were grouped under the “standard dose VTE prophylaxis. Any patient who received higher than standard dose but not as treatment dose (Enoxaparin 1 mg/kg q12hr or 1.5 mg/kg q24hr or UFH infusion) was categorized as receiving “High VTE prophylaxis dose”. On the other hand, lower VTE prophylaxis considered for patient who received Enoxaparin < 40 mg/day or Unfractionated heparin (UFH) < 5000 Units three times daily/day)

### Multiple organ dysfunction (MODS)

Details of the MODS on day three are shown in (Table 2). Best GCS, CVP, heart rate, pressure adjusted heart rate, and PaO2/FiO2 were significantly different between the two groups. The PaO2/FiO2 on day three of ICU admission was higher in the methylprednisolone group than dexamethasone; however, it was higher at baseline (Table 1).﻿ In crude analysis, the MODS on day three of ICU was higher in patients who received methylprednisolone when compared to dexamethasone. Similarly, at regression analysis, patients in the methylprednisolone group have a higher MOD score (beta coefficient: 0.17 (95% CI 0.02, 0.32), *P* = 0.03) (Table 3).

**Table 2:** Details of the multi-organ dysfunction on day 3

Outcomes	Dexamethasone	Methylprednisolone	*P*-value
Best GCS (Without sedation) at day#3, Median (Q1,Q3)	15.0 (9.00, 15.00)	10.5 (3.00, 15.00)	0.0025^^^
CVP at day#3, Median (Q1,Q3)	8.0 (8.00, 8.00)	8.0 (8.00, 12.00)	0.0318^^^
Heart rate at day#3, Median (Q1,Q3)	75.0 (62.00, 89.00)	88.0 (76.00, 100.00)	0.0001^^^
Pressure adjusted heart rate at day3, Median (Q1,Q3)	9.0 (7.32, 11.43)	10.9 (8.41, 15.69)	0.0003^^^
MAP at day#3, Median (Q1,Q3)	73.0 (65.00, 82.00)	70.0 (63.00, 81.00)	0.2446^*^
PaO2/FiO2 (Lowest) at day3, Median (Q1,Q3)	120.8 (80.50, 190.00)	145.0 (100.00, 235.50)	0.0311^^^
Platelets count (Lowest) at day3, Median (Q1,Q3)	261.0 (202.00, 338.00)	300.0 (199.00, 375.00)	0.0836^^^
Serum creatinine (Highest) at day3, Median (Q1,Q3)	84.0 (66.00, 130.00)	96.0 (68.00, 196.00)	0.1386^^^
Total Bilirubin (Highest) at day3, Median (Q1,Q3)	9.0 (6.70, 13.00)	10.8 (6.90, 17.80)	0.2434^^^

^*^T –Test

^^^Wilcoxon rank sum test is used to calculate the *P*-value

**Table 3:** The outcomes of critically ill patients with COVID-19 after propensity score matching

**Outcomes**	**Number of outcomes/Total number of patients**			**Hazard Ratio (HR) (95%CI)**	***P*-value**^$^
	**Dexamethasone**	**Methylprednisolone**	***P*-value**		
**30-day mortality, n (%)**	62 (34.8)	25 (41.7)	0.3417^^^^	0.997 (0.970, 1.025)	0.85
**In-hospital mortality, n (%)**	64 (35.6)	29 (46.8)	0.1173^^^^	1.02 (1.0, 1.04)	0.05
				**beta coefficient (Estimates) (95%CI)**	***P*-value**^$*^
**MODS at day 3 of ICU admission, Median (Q1,Q3) ∆**	5.0 (3.00, 8.00)	6.0 (4.00, 9.00)	0.1151^^^	0.17 (0.02,0.32)	0.03
**VFDs, Median (Q1,Q3)**	18.0 (0.00, 27.00)	14.0 (0.00, 27.00)	0.4849^^^	-0.12 (-0.62,0.39)	0.65
**ICU Length of Stay (Days), Median (Q1,Q3)**	10.0 (6.00, 18.00)	10.0 (7.00, 18.00)	0.7702^^^	0.06 (-0.14,0.25)	0.56
**Hospital Length of Stay (Days), Median (Q1,Q3)**	17.0 (11.00, 26.00)	19.0 (13.00, 27.00)	0.6576^^^	-0.15 (-0.35,0.05)	0.14

^^^Wilcoxon rank sum test is used to calculate the *P*-value

^^^^Chi-square test is used to calculate the *P*-value

^$^Time dependent cox proportional hazards regression analysis used to calculate HR and *p*-value

^$*^Generalized linear model is used to calculate estimates and *p*-value

### Complications during ICU stay

**Fig. 2:**
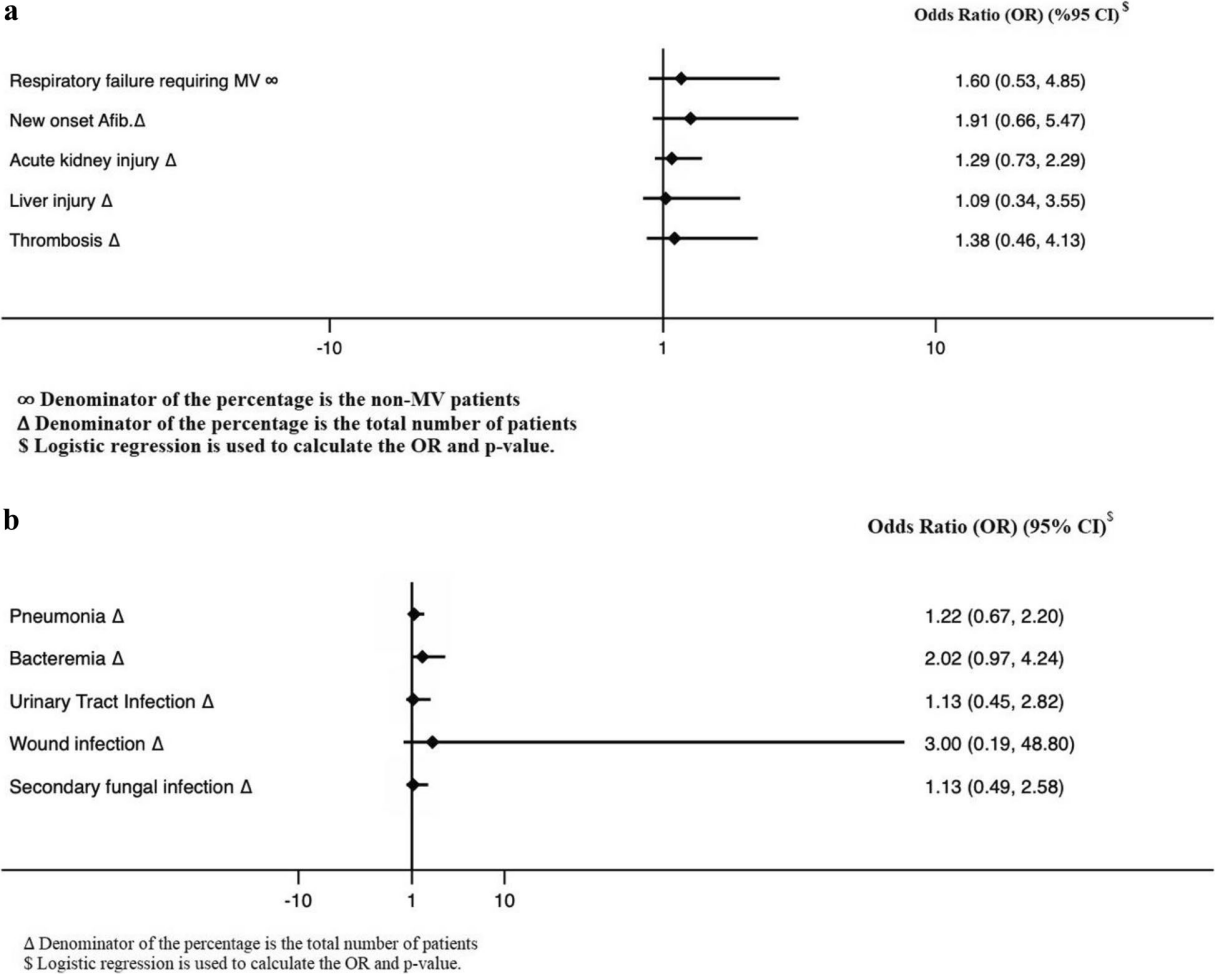
**a** Complications during ICU stay. **b** Infection complications during ICUs

Among the non-MV patients within 24 h of admission, patients who received methylprednisolone have a higher odd of respiratory failure that requires MV; however, it was not statistically significant (OR 1.60, 95% CI 0.53, 4.85; *p* = 0.41). Patients who received methylprednisolone had a higher odds of infection complications during ICU than dexamethasone; however, didn’t reach a statistically significant difference. Moreover, other complications during the stay, such as new-onset atrial fibrillation, AKI, and liver injury, were similar between the two groups (Fig. 2a, b).

### 30-day and in-hospital mortality

The prevalence of 30-day mortality in the crude analysis was 34.8% in the dexamethasone group compared with 41.7% in patients who received methylprednisolone, which was not statistically significant (*P* = 0.34). Similarly, multivariable time dependent cox proportional hazards regression analysis, revealed a similar 30-day and in-hospital mortality (HR 0.997, 95% CI 0.97, 1.03; *p* = 0.85 and HR 1.02 95% CI 1.0, 1.04; *p* = 0.053 respectively) (Table 3).

### Ventilator free days and length of stay

The median VFDs was 18.0 (0.0, 27.0) days in the dexamethasone group compared with 14.0 days in patients who received Methylprednisolone (*P* = 0.48). In addition, the difference was not statistically significant at regression analysis (beta coefficient: -0.12 (95% CI -0.62, 0.39); *P* = 0.65). Moreover, there was no statistically significant differences in the ICU and hospital length of stay (beta coefficient: 0.06 (95% CI -0.14, 0.25); *P* = 0.56 and beta coefficient: -0.15 (95% CI -0.35, 0.05); *P* = 0.14, respectively) (Table 3).

## Discussion

This retrospective multicenter study compared clinical and safety outcomes between dexamethasone and methylprednisolone among 264 critically ill patients with COVID-19. After PS matching and linear regression model analysis, we found a statistically significant lower MODS among patients treated with dexamethasone compared to methylprednisolone. Similar findings were reported after adjusting for age and MODS within 24 h of ICU admission. Indeed, our study found that patients treated with methylprednisolone had significantly lower GCS, higher PaO_2_/FiO_2_, and pressure-adjusted heart rate. One possible explanation for higher PaO_2_/FiO_2_ is the well-established higher lung tissue-to-plasma ratio of methylprednisolone [26–28]. Although is it controversial, it has been considered that dexamethasone penetrates the blood–brain barrier relatively more than other corticosteroids [29–31]. This could explain the significantly higher GCS scores among patients treated with dexamethasone in our study. It is important to note that GCS assessment in invasive MV is limited by the uncertainty surrounding GCS assessments in the context of intubation. The CoDEX trial investigated the effect of dexamethasone on COVID-19 patients and concluded that dexamethasone resulted in a significantly higher number of free-from MV days than the standard of care alone despite the similar PaO_2_/FiO_2_ ratios of both groups [32]. Furthermore, in the RECOVERY trial, dexamethasone-treated patients had a lower need for renal-replacement therapy and invasive MV [16]. Despite using a lower dexamethasone dose compared to CoDEX trial and having a higher CRP and lower PaO2/FiO2 ratio at admission, our dexamethasone-treated patients had lower MODS [32].

Regarding secondary outcomes in our study, our study showed a higher odds of infection complications during ICU among methylprednisolone-treated cohort than dexamethasone; however, didn’t reach to a statically significant difference. A large meta-analysis suggests higher secondary infections following corticosteroid use among influenza patients [33]. However, similar findings were not observed in other studies that mainly looked at COVID-19 patients [34]. The Metcovid trial included suspected and confirmed cases of COVID-19 and investigated the effect of methylprednisolone on mortality [35]. The similar rates of hospital-acquired infection in the Metcovid study could be explained by the absence of use of other immunomodulator COVID-19 therapies. In our study, a major factor could have contributed to the higher hospital-acquired infection rate with methylprednisolone. Methylprednisolone is a relatively more potent immunosuppressant than dexamethasone [35]. Although it was statistically non-significant, patients treated with methylprednisolone had higher ICU and hospital lengths of stay. Several studies showed longer hospital and ICU lengths of stay result in a higher infection rate [36, 37].

Most importantly, the higher MODS and infection rates were not translated into a higher mortality rate with methylprednisolone than dexamethasone. While we did not statistically adjust for SOFA scores or age, the relatively young patient population and low predicted mortality rate based on SOFA scores among our patients could have impacted the mortality rates in our analysis. Similarly, other studies demonstrated a reduction in 28-days and in-hospital mortality [10, 12–14]. Three of the studies mentioned included more elderly patients and had used more immunomodulators [10, 12, 13]. Another study included patients with much lower CRP than ours, indicating a less acute COVID-19 inflammation [14]. These factors could be attributed to the lower mortality rates in other studies. In contrast to other studies, we reported similar outcomes between methylprednisolone and dexamethasone on ventilator-free days and respiratory failure requiring MV [10, 12, 34]. The low need for MV at admission and lower ICU admission rates in the previously published studies might explain the differences in our findings.

Our study has a few limitations, including its retrospective nature which limit capturing some important variables such a; medications used prior to ICU admission, as well as onset of complications such as infections and relatively small sample size. In addition, our primary outcome MODS was only assessed at day three which limits the evaluation of the variation in MODS scores between the two groups over a continuous period. On the other hand, this study has some strengths, including multicenter patient recruitment, and PS matching was used to eliminate a greater portion of bias and create a balanced dataset.

## Conclusion

This study showed a lower MODS on day three of ICU admission in critically ill patients with COVID-19 treated with dexamethasone. However, both interventions had a similar mortality rate. Future RCTs are needed to confirm our findings and to assess MODS over an extended period during ICU stay.

### Supplementary Information

**Additional file 1.** Outcome definition (s).

## Data Availability

The datasets used and/or analyzed during the current study are available from the corresponding author upon reasonable request.
