# Supplementary material for: Dexamethasone versus methylprednisolone for multiple organ dysfunction in COVID-19 critically ill patients: a multicenter propensity score matching study
Source: BMC Infect Dis. 2024 Feb 13;24:189. doi: 10.1186/s12879-024-09056-y (PMC10863167; doi:10.1186/s12879-024-09056-y)
Supplement: Supplementary file 1 — Additional file 1. Outcome definition (s). [file 12879_2024_9056_MOESM1_ESM.docx]

**Additional file 1: Outcome definition (s)**

- (MODS) is used to measure the severity of the multiple organ dysfunction syndromes in six organ systems: 1) the respiratory system (Po2/FIO2 ratio); 2) the renal system (serum creatinine concentration); 3) the hepatic system (serum bilirubin concentration); 4) the hematologic system (platelet count); 5) the central nervous system (Glasgow Coma Scale (GCS)), and 6) the pressure-adjusted heart rate (heart rate multiplied with the ratio of central venous pressure (CVP)to mean arterial pressure(MAP))^1^.
- The 30-day mortality was defined as a death occurring for any cause within 30 days of admission. Patients who were discharged from the hospital alive were presumed to be survived. All patients were followed until they were discharged from the hospital or died during the in-hospital stay, whichever occurred first.
- (VFDs) at 30 days were calculated as follows: VFDs = 30 − days after MV initiation (if the patient survived and was successfully liberated from MV). If the patient died within 30 days of MV, or if the patient was on MV for > 30 days, the VFDs = 0.
- (AKI) was defined as a sudden decrease of renal function within 48 hours. An increase in absolute SCr of at least 26.5 μmol/L (0.3 mg/dL) or by a percentage increase in SCr ≥ 50% (1.5× baseline value) during ICU stay ^2^.
- Acute liver injury was defined as alanine aminotransferase (ALT) exceeding three times the upper limit of normal or double in patients with elevated baseline ALT during the ICU stay ^3^.
- Secondary fungal infection was identified through the blood, urine, wound, drainage, cerebrospinal fluid, and/or respiratory cultures. Cultures were excluded if the laboratory reported them as a "contaminant sample." The fungal growth was considered significant if the growth was ≥ 100,000 colony forming units (CFUs)/ml in sputum or endotracheal aspiration, ≥10,000 CFUs of single organism/ml in bronchoalveolar lavage, or ≥ 1000 CFUs of single organism/ml in protected specimen brushes. Additionally, urinary cultures were considered significant if showing a growth ≥100,000 CFUs/ml of no more than two species of microorganisms ^4^.
- Respiratory failure was defined as having a low arterial carbon dioxide tension (PaCO_2_) or hypoxemic respiratory failure (PaO_2_ < 60 mm Hg) with a normal or hypercapnic respiratory failure (PaCO_2_ > 50 mm Hg) that requires MV ^5^.

**References**

1. Marshall, J. C. *et al.* Multiple organ dysfunction score: a reliable descriptor of a complex clinical outcome. *Crit. Care Med.* **23**, 1638–1652 (1995).

2. Lin, C.-Y. & Chen, Y.-C. Acute kidney injury classification: AKIN and RIFLE criteria in critical patients. *World J. Crit. care Med.* **1**, 40–45 (2012).

3. Da, B. L. *et al.* Liver Injury in Hospitalized Patients with COVID-19 Correlates with Hyper Inflammatory Response and Elevated IL-6. *Hepatol. Commun.* **5**, 177–188 (2020).

4. Aleidan, F. A. S. *et al.* Incidence and risk factors of carbapenem-resistant Enterobacteriaceae infection in intensive care units: a matched case-control study. *Expert Rev. Anti. Infect. Ther.* **19**, 393–398 (2021).

5. Rodriguez-Roisin, R. Pulmonary gas exchange in acute respiratory failure. *European Journal of Anaesthesiology* vol. 11 5–13 https://pubmed.ncbi.nlm.nih.gov/8143714/ (1994).
